# Spatially Resolved Uncertainties for Machine Learning Potentials

**DOI:** 10.1021/acs.jcim.4c00904

**Published:** 2024-08-07

**Authors:** Esther Heid, Johannes Schörghuber, Ralf Wanzenböck, Georg K. H. Madsen

**Affiliations:** Institute of Materials Chemistry, TU Wien, A-1060 Vienna, Austria

## Abstract

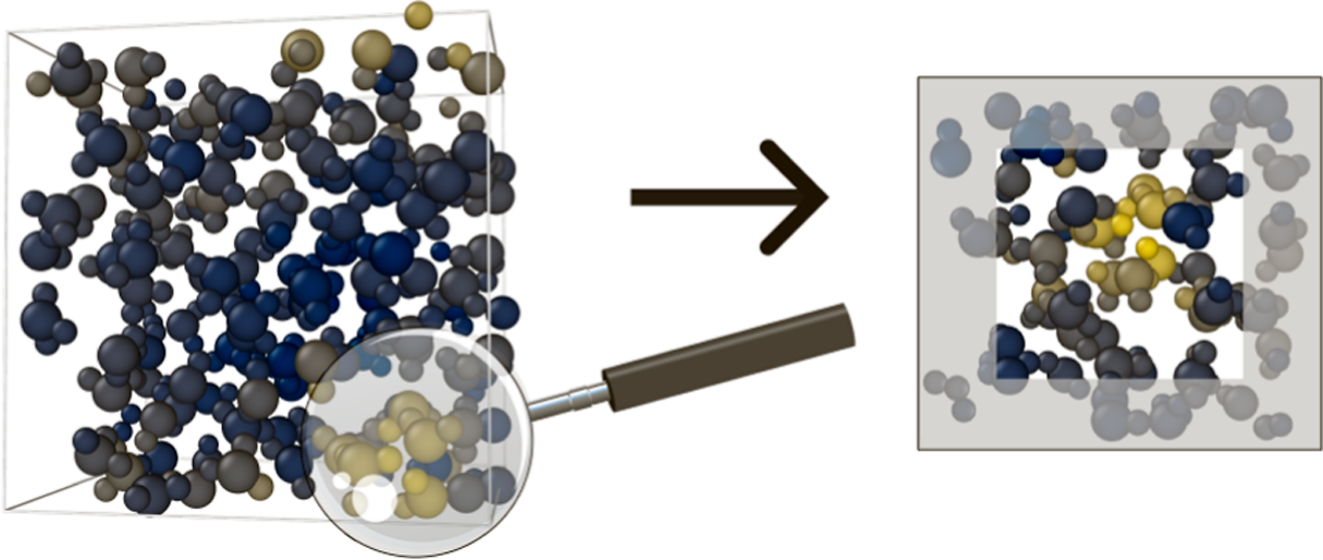

Machine learning potentials have become an essential tool for atomistic simulations, yielding results close to ab initio simulations at a fraction of computational cost. With recent improvements on the achievable accuracies, the focus has now shifted on the data set composition itself. The reliable identification of erroneously predicted configurations to extend a given data set is therefore of high priority. Yet, uncertainty estimation techniques have achieved mixed results for machine learning potentials. Consequently, a general and versatile method to correlate energy or atomic force uncertainties with the model error has remained elusive to date. In the current work, we show that epistemic uncertainty cannot correlate with model error by definition but can be aggregated over groups of atoms to yield a strong correlation. We demonstrate that our method correctly estimates prediction errors both globally per structure and locally resolved per atom. The direct correlation of local uncertainty and local error is used to design an active learning framework based on identifying local subregions of a large simulation cell and performing ab initio calculations only for the subregion subsequently. We successfully utilized this method to perform active learning in the low-data regime for liquid water.

## 1 Introduction

In recent years, machine learning potentials have gained importance as data-driven energy and force predictors for atomistic simulations, achieving an accuracy close to ab initio results while offering a considerable speedup. The underlying model architectures have improved from neural networks built on simple invariant encodings of the atom environment^1−3^ to elaborate equivariant, possibly multibody interactions based on graph neural networks^4−7^ or graph transformers^8^ allowing both more precise and more data-efficient models.

With excellent models at hand, the focus has now shifted to the quality and quantity of the underlying data. New data sets such as the Open Catalyst 2020 and 2022 data sets^9,10^ for adsorbates on surfaces, ANI-1x^11^ for organic molecules, or Transition1x^12^ for simple organic reactions have emerged and can be used to train a baseline model, which can subsequently be fine-tuned to a system of interest. Since ab initio calculations are costly, it is nevertheless essential to develop clever data generation strategies, i.e., to identify where a model fails so that new ab initio calculations can be issued and added to the training data. The quantification of the estimated error in a prediction is, furthermore, essential for decision-making processes. For atomistic simulations, the force uncertainty is key to determine whether the simulation is exploring structures that the machine learning potential is confident about.^13,14^

The identification of high-error predictions is still an open challenge in machine learning across many fields of research, and viable approaches depend on the details of the data set, task, and model architecture. The error in a model prediction can be dissected into aleatoric (irreducible by addition of data) and epistemic (reducible by addition of data) contributions.^15−19^ The aleatoric contribution stems from noise in the input data or missing input features and can be learned by the model itself using heteroscedastic loss models (mean variance estimation) and variations thereof,^20,21^ or can be estimated posthoc.^22,23^ The epistemic contribution is associated with a limited knowledge of the model, which can be further dissected into error from model variance and model bias. It is usually approximated by training a committee of models, varying the model initialization seed, hyperparameters, architecture, or training data and monitoring their disagreement on a prediction to obtain a proxy for the error from model variance.^24−26^ Other techniques furthermore obtain a combined measure for aleatoric and epistemic error.^27,28^ Yet, especially, epistemic error is notoriously difficult to model, since the above approaches only capture error from variance but not from model bias. Yet, model bias can be the major source of error in a model especially for small data sets and difficult-to-learn targets, so that the epistemic uncertainty obtained from committees often underestimates the model error.^19,29^

In the field of machine learning potentials, uncertainty estimation techniques have achieved mixed results.^21,30−33^ Here, aleatoric uncertainty is usually negligible, since there is a direct, learnable relation between the input (the atomic numbers and coordinates) and the target (the ab initio energies and forces) if different spin states and magnetic states are not taken into account. However, there is no direct correlation between the epistemic uncertainty of a single data point obtained from the standard deviation of the committee predictions and the absolute error. This behavior has been reported for a wide variety of data sets and model architectures,^30,32,34−36^ but its origin and possible remedies have not been identified yet. Heuristic approaches to average force uncertainties over structures as a proxy for model error have achieved success in active learning settings^31,37^ but are missing a theoretical framework and explanation of why and when such an approach is recommendable. Moreover, as simulations based on machine learning potentials are moving toward trillions of atoms^38^ and ab initio calculations become infeasible for the full system, even a perfect estimator of the overall error is insufficient. For active learning, it is therefore essential to trace down the overall error and uncertainty to smaller regions within a system, which can then be isolated. While recent approaches to active learning have attributed the uncertainty on a per atom basis,^39−41^ there is no guarantee that the obtained atomic uncertainties actually correlate with the model error. In fact, recent studies reported that there is no direct correlation between the uncertainty of atomic forces and the actual error in the force prediction of that atom.^30,31^

In summary, the unresolved challenges of accurate global and local uncertainty estimates largely hinder the development of efficient active learning cycles for machine learning potentials. Yet, the field has a major advantage over other prediction tasks: Instead of learning to predict a single quantity (one target value per data point), a machine learning potential always predicts a molecular/full-structure energy as well as atomic forces for each atom in each spatial direction. In the current study, we discuss why approaches based on aggregating uncertainties over all atoms in a data point give reliable uncertainty estimates, providing a theoretical framework for previous works.^31,37^ We then take the concept a step further and present a new, model-agnostic, simple, and fast method to obtain spatially resolved uncertainties that correlate with the actual error for all atoms within a data point. We detail the theoretical basis of our approach and demonstrate it on diverse systems, namely, organic reactions in the gas phase, perovskite structures, and liquid water, where we find that global and spatially resolved local model errors can be predicted quantitatively. We then show how our approach enables a reliable identification of spatially resolved high-error regions using simple committee standard deviations and demonstrate the capabilities of these local uncertainties for active learning.

## 2 Methods

### 2.1 Data Sets and Models

The equivariant message-passing neural network MACE^4^ was used as provided, with hyperparameters as indicated in the following paragraphs. In all cases, training was performed using the AMSGrad optimizer with hyperparameters and learning rate schedules given by the defaults set in the MACE package. All models were constructed using two layers with 128 channels for even and odd parity features and a maximum order *l*_max_ = 3 of spherical harmonics. Eight Bessel basis functions and a polynomial cutoff function of order *p* = 5 were used for generating the radial features, which are passed into MLPs with three layers of 64 nodes each and SiLU serving as the nonlinear transfer function. The final readout function generating the atomic energies is given by an MLP with a single layer of 16 hidden features. In the Supporting Information, we furthermore report results with smaller MACE models (differing in the number of channels and cutoff radius), as well as invariant NeuralIL^2,31^ models.

Transition1x^12^ was downloaded as provided. From the roughly 10 M data points of 10,073 reactions, we only kept the last, converged reaction pathways, where each pathway is made up of 10 images of the nudged elastic band (NEB) search, resulting in 100 k data points. The data set was then split into a training and validation fraction made up of structures of the first or last two images (index 1, 2, 9, and 10) in the NEB search (40 k data points), corresponding to the equilibrated reactants, products, and configurations close to these equilibrated structures. All other image indices were put into the test set (60 k data points). This split allows us to explore the correlation of epistemic uncertainty with the absolute error for regions the model has never seen, namely, nonequilibrium configurations along diverse reaction paths. Since the image indices are known for all data points, we can evaluate the correlation as a function of the distance to the training image indices and therefore explore mild (index 3 and 8) to strong (index 5 and 6) out-of-distribution examples.

MACE models for Transition1x were trained with a cutoff of 5 Å for a maximum of 1400 epochs with an early stopping patience of 50, with a force weight of 100.0 and an energy weight of 1.0. Then, 100 further epochs without early stopping were conducted with a force weight of 100.0 and an energy weight of 1000.0.

SrTiO_3_(110)-4 × 1 structures were obtained from a subset of structures originally published in ref (42), which were then re-evaluated via VASP version 6.2.0^43^ single-point evaluations with the r^2^SCAN functional.^44^ The energy cutoff was set to 440 eV and the width of Gaussian smearing was set to 0.02 eV. The final data set contained 889 unique structures, which were split randomly into training, validation, and test sets with 554, 237, and 98 data points, respectively.

MACE models for SrTiO_3_ were trained with a cutoff of 4 Å for a maximum of 1200 epochs with an early stopping patience of 50, where the force and energy weights corresponded to 100.0 and 1.0, respectively. Subsequently, 300 epochs without early stopping were conducted at force and energy weights of 100.0 and 1000.0, respectively.

Finally, 1593 structures of 64 water molecules originally calculated at the revPBE0-D3 level of theory and periodic boundary conditions were taken from Cheng et al.^45^ as provided. Since the set contains five structures with duplicate atomic positions, these were removed from the data set, resulting in 1588 unique structures. The energies and forces for these structures were recomputed at the RPBE-D3^46,47^ level of theory using VASP version 6.4.2.^43^ The hard PAW potential setups provided by the VASP package were used, with the cutoff energy set to 850 eV, the width for Gaussian smearing set to 0.05 eV, and solely the Γ-point of the Brillouin zone being sampled. Following the results reported in ref (48), D3 corrections have been computed with the zero-damping scheme. The data set was randomly split into training, validation, and test sets with ratios 80:10:10.

MACE models for liquid water were all computed with a cutoff radius of 4 Å. Training was initially run with a force weight of 100.0 and an energy weight of 1.0 for 800 epochs without early stopping. Subsequently, the energy and force weights were adjusted to 1000.0 and 100.0, respectively, and 200 more epochs were performed.

### 2.2 Active Learning for a Molecular Dynamics Simulation of Water

Three independent active learning cycles were run for 15 iterations: one based on identifying new structures using local uncertainties, one based on atomic uncertainties, and one based on sampling randomly. All start from a model trained on an 80:20 split of the 50 highest energy structures contained in the water data set described above. At each iteration, a 20 ps molecular dynamics (MD) simulation of a system containing 128 water molecules at a fixed density of 0.86 g cm^–3^ was run with a time step of 0.5 fs in the *NVT* ensemble at 350 K using a Nosé–Hoover thermostat. LAMMPS version 2023.3.28^49^ was used with MACE as the engine for energy and force evaluations. The given density lies below the experimental density of water, as expected for the RPBE-D3 level of theory.^48^

The trajectories obtained from the MD simulations were used to generate new training data to improve the models: At each active learning step, the first 10 ps of the trajectory were divided into ten evenly sized segments to guarantee that new structures were sampled at different intervals of the simulation. For the active learning cycle based on randomly selecting new data, a random snapshot was selected from each of the ten segments. For the local uncertainty-based runs, snapshots were selected from the subsets by calculating the locally aggregated uncertainties (as detailed in the Results section) for each atom based on a committee of five MACE models using an aggregation *r*_cut_ = 4 Å and choosing the frames featuring the environments with the highest uncertainties. For the atomic uncertainties, frames and atoms were selected based on the highest atomic force uncertainty (as detailed in the Results section). Boxes containing 64 water molecules were subsequently cut from the snapshots: A central oxygen atom and the 63 closest oxygen atoms to it were selected, and the water molecules were determined by selecting the two hydrogen atoms closest to each oxygen, respectively. The box length for the new, smaller configuration is given by  and the originally selected center oxygen atom was placed in the center of a cubic box with this length. For the active learning cycle based on local uncertainties, the central atom was given by the oxygen atom featuring the maximum local uncertainty in the snapshot. For the run based on random selection, the index of the central oxygen atom was randomly generated. To avoid introducing high energies and forces due to cutting a periodic box from a larger initial configuration, the atoms close to the border of the new box were relaxed by using the MACE model at the current active learning iteration based on the following procedure: All atoms within a distance of 0.8*l*_new_/2 of the central oxygen atoms were kept fixed. The box was then padded with 2 Å in each direction. Five BFGS iterations were performed to relax the positions of the free atoms, and the box size decreased by 0.2 Å in each direction. This procedure was repeated until the original box size was recovered to allow the border regions to relax to physically meaningful structures. Energies and forces for the configurations obtained by this procedure were then calculated by using the DFT setup described above. A new active learning iteration was then started by adding the new configurations to the data set from the previous iteration and retraining MACE models from randomly initialized model parameters based on the new data set.

We furthermore generated ten additional data points per AL cycle for both random and local uncertainty-based selection following the exact same procedure as described above, utilizing the trajectory from 10 to 20 ps. This data was used as an independent test set, totaling 360 data points.

## 3 Results

### 3.1 Benchmark Model

In this subsection, we detail the general method developed in this study and verify it by the Monte Carlo simulation of a simple system featuring model variance as its only error source. In the following, we then discuss real data sets where model bias can play a significant role.

Due to the large flexibility of neural networks, training a model multiple times from different starting configurations yields slightly different predictions which can be assumed to approximately follow a Gaussian distribution. The following results will therefore be derived under the assumption that the committee predictions follow such a distribution. We first consider a committee of *N*_*C*_ independently trained models with model variance as the only source of error. The committee will provide *N*_*C*_ predictions  (with *l* = 0, 1, ..., *N*_*C*_), with mean *ŷ* and the committee standard deviation
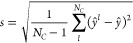
1computed as the unbiased estimator of the population standard deviation. In the following, we compute *s* as defined above, but note that for a low number of committee members, a correction to the underestimation of the true population standard deviation by the committee standard deviation can be applied.^50^ The mean is subsequently used as overall model prediction and *s* as an estimator of the model uncertainty. Repeating this experiment (i.e., training *N*_*C*_ models and averaging their predictions  to *ŷ*) multiple times would reveal that *ŷ* is distributed around the target with a standard deviation of . Choosing the target *y* = 0, we can, without loss of generality, then obtain the expectation value of the absolute error via
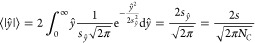
2

While eq 2 shows that the absolute error, on average, is related to the committee standard deviation by a factor , this is only true for a large number of repeated experiments of committee training and predictions. For a single committee prediction *ŷ*, for example, predicting the molecular energy or the force of a single atom, the model error |*ŷ*| cannot be directly correlated with the uncertainty obtained from the committee standard deviation *s*, because the prediction corresponds to a single random draw from the underlying distribution. At the same time, a large number of repeated committee trainings are prohibitively expensive in practice, so that the correlation of the absolute error and the committee uncertainty through eq 2 might seem inapplicable to machine learning. Alternatively, the conversion factor from eq 2 can also be recovered by averaging over targets within a data set of *N*_*i*_ data points, instead of averaging over multiple experiments of model retraining and prediction for a single target. In this case, the mean absolute error over all targets *y*_*i*_ is related to the mean committee standard deviation by a factor 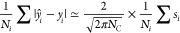
3

This is frequently used to compute, e.g., error calibration curves.^36,51^ Nevertheless, averaging over data points is undesirable, since we aim for a direct correlation of error and uncertainty for a single data point.

Machine learning potentials, however, yield not only a prediction of a single energy value per configuration but also forces for each atom. Thus, each data point *i* in a data set of size *N*_*i*_ consists of 3*N*_*j*_ force components, where *N*_*j*_ is the number of atoms. As a result, the model predictions  aim to reproduce the target forces *f*_*ij*_^*k*^ with *k* denoting the spatial direction. The predicted forces  are obtained as a committee average over *N*_C_ committee predictions
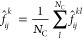
4resulting in a prediction error of
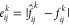
5and a committee standard deviation of
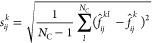
6

Similar to the prediction of a single target discussed above, *e*_*ij*_^*k*^ and *s*_*ij*_^*k*^ cannot be correlated per atom, because again a single committee prediction corresponds to a single draw from a distribution centered around the target with a width determined by . However, we can utilize force predictions averaged over sets of atoms to correlate the variance error with uncertainty. Namely, by averaging over all directions *k* and atoms *j*, we arrive at a per-structure mean absolute error of
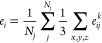
7and an average standard deviation of
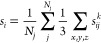
8

**Figure 1:**
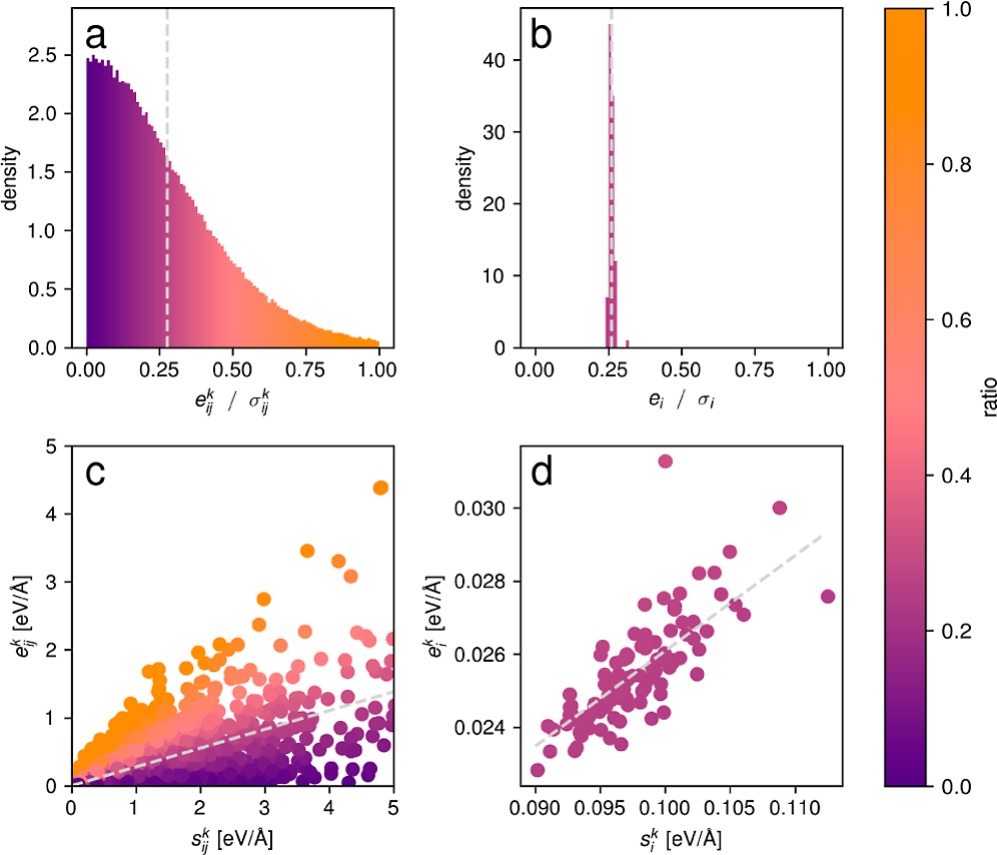
Histogram of the ratio of the absolute error and uncertainty for Monte Carlo committee predictions featuring model variance as the only error source for (a) individual atoms of all data points and (b) aggregated values within each data point. The gray dashed line indicates the average over all data points. The individual combinations of errors and uncertainties are furthermore shown (c) per atom or (d) per data point, colored according to the error/uncertainty ratio. The gray dashed line indicates the fit obtained via a mean α. The data were obtained with the parameters *N*_*C*_ = 10, *N*_*i*_ = 100, *N*_*j*_ = 1000, and ground-truth uncertainties drawn from an inverse-Gamma distribution.

In the following, we will use Monte Carlo simulation to corroborate that *e*_*i*_ and *s*_*i*_ are strongly correlated whereas *e*_*ij*_^*k*^ and *s*_*ij*_^*k*^ are not. For a data set of *N*_*i*_ structures consisting of *N*_*j*_ atoms each, we draw σ_*ij*_^*k*^ from an inverse-Gamma distribution as proposed in ref (28), so that each combination of *i*, *j*, and *k* gets assigned its own ground-truth uncertainty. We then draw from normal distributions with μ = 0 and σ = σ_*ij*_^*k*^ to obtain *N*_C_ model predictions of a committee for each combination of *i*, *j*, and *k*. Subsequently, we compute the committee standard deviation *s*_*ij*_^*k*^ and absolute error *e*_*ij*_^*k*^ as well as the averaged values *s*_*i*_ and *e*_*i*_ within a data point (eqs 7 and 8) from the committee predictions. The simulation thus corresponds to a simple artificial system, where an otherwise perfect model features only variance error. Figure 1 depicts histograms of the error-uncertainty ratio for the individual and aggregated case as well as scatter plots of the errors vs uncertainties. In the Supporting Information, we furthermore report error-uncertainty ratios as a function of the size of the committee. For *N*_*C*_ = 10, the conversion factor between error and uncertainty from eq 3 is . Clearly, both individual and aggregated versions converge to a ratio of 0.25 averaged over all data points (gray dashed lines). However, as expected,^30,32,34−36^ the individual error-uncertainty ratios are broadly distributed so that there is no linear dependency between error and uncertainty for the individual data points, as visible in Figure 1c. Figure 1a,c furthermore depicts that the distribution of the predicted values around the target leads to more predictions being observed toward the center of the distribution (small error, dark purple) than its outskirts (orange). Therefore, many individual uncertainty values obtained from the committee standard deviation significantly underestimate the actual model error, so that the individual committee uncertainties cannot be used to identify high-error atoms. The relation between atomic uncertainties and errors is therefore asymmetric, with large errors occurring only for large uncertainties, and small uncertainties permitting only small errors, but not the other way around.^34^ In contrast, the ratio of averaged absolute errors over the uncertainties shows a narrow distribution, as visible in Figure 1b,d, so that we can actually estimate the prediction error from the uncertainty by multiplication with 0.25, corroborating eq 3. Note that the conversion factor for this artificial system being lower than 1.0 results from the model being unbiased, which is usually not the case for real atomic systems.^30,31^

Monte Carlo simulations were performed for different *N*_C_, *N*_*i*_, and *N*_*j*_, as well as different distributions for σ_*ij*_^*k*^, namely, uniformly random distributions within an interval, normal distributions, and even constant values, and the same behavior was observed across all systems: The individual ratios are centered around  but are distributed too broadly to show a meaningful correlation between the absolute errors and uncertainties, while the ratio of the respective aggregated values is distributed in a narrow peak yielding a strong correlation.

We can therefore conclude that for models where the variance is the main source of error, the mean force error over a structure or molecule can be easily predicted by multiplication of the mean committee standard deviation by . For cases where model variance is the only source of error, we have thus identified a method to correlate the error and uncertainties within a single data point by aggregating over the errors and uncertainties of all atoms within that data point. In the following, we explore whether this method is also applicable to models featuring bias errors of different magnitudes.

### 3.2 Real Systems with Mixed Error Sources

In addition to model variance, nearly all machine learning models also suffer from model bias, which can stem from fundamental shortcomings of the model, too little data, or ill-chosen features, among others.^29^ Uncertainty estimates obtained from an ensemble of models capture only variance errors. Since model bias, as introduced due to the aforementioned reasons, can be the dominant contribution, ensemble uncertainties usually underestimate the actual error.^30,31^ We therefore examine whether our approach also holds up for machine learning potentials trained on real data sets.

Since we aggregate force uncertainties over atoms, the number of atoms per data point is important and we chose three data sets examining a large variety of system sizes and configurations. (1) Transition1x^12^ features NEB searches for a wide range of organic reactions in the gas phase with 7 to 23 H, C, N, and O atoms and thus resembles rather small systems without periodic boundary conditions. From each reaction, we only used the last, converged reaction pathways made up of ten images each, where indices 1, 2, 9, and 10 were used as the training and validation set. The test set can thus be split into different indices corresponding to a different level of extrapolation from the training configurations. (2) Surface reconstructions of crystalline SrTiO_3_ were taken from ref (42) and feature 136 Sr, Ti, and O atoms per data point, thus resembling large systems with periodic boundary conditions. (3) A liquid water data set from ref (45) features 192 H and O atoms per data point and thus resembles a large, homogeneous system with periodic boundary conditions that lends itself to dissecting the overall simulation cell into smaller subregions. On each data set, we trained a five-member ensemble of the equivariant message-passing neural network MACE.^4^ See the Methods section for further details.

**Figure 2:**
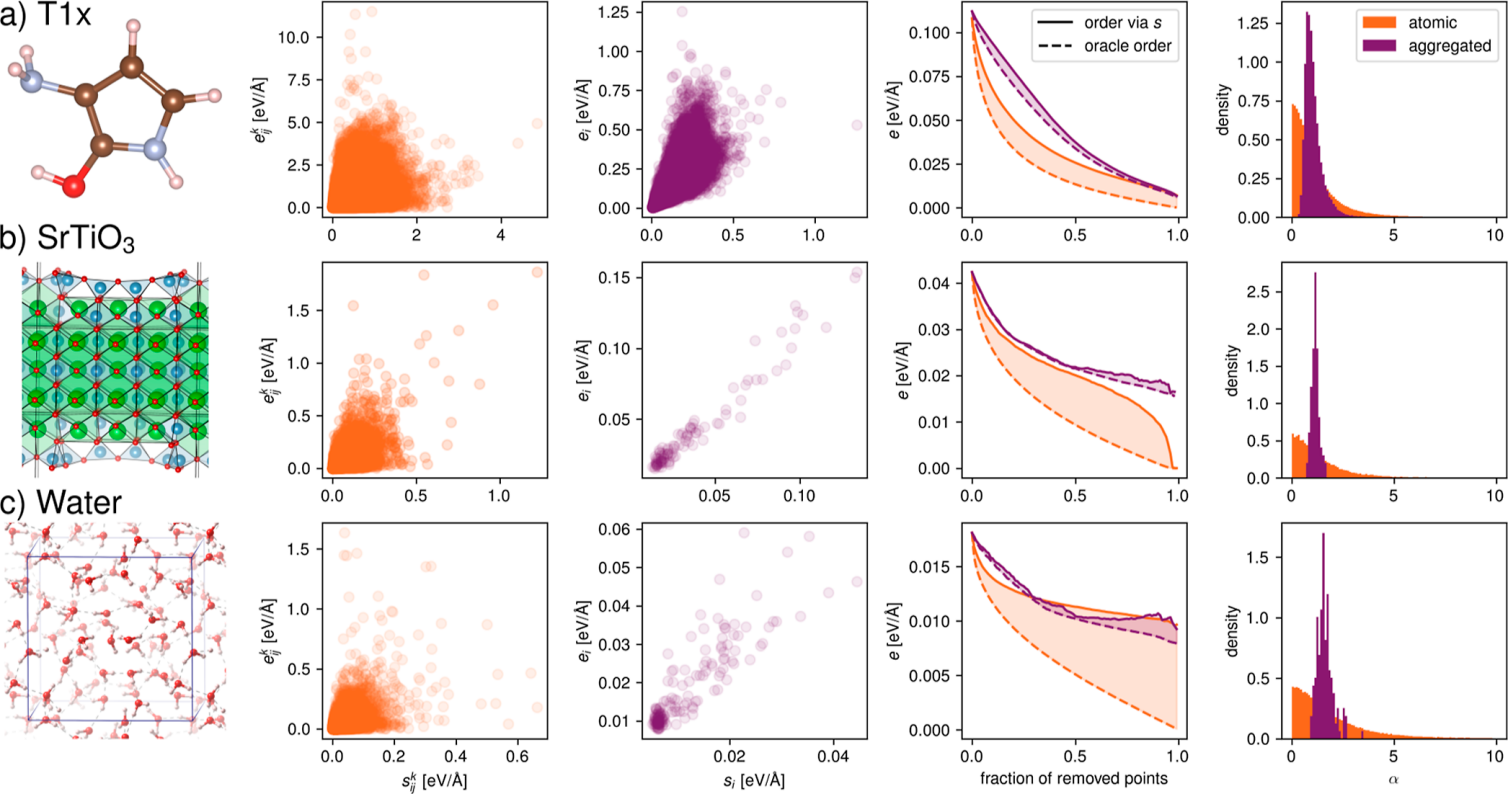
Relation between the absolute error and uncertainty for the Transition1x data set (a, top row), the SrTiO_3_ data set (b, middle row), and the liquid water data set (c, bottom row). For each data set, individual absolute errors vs uncertainties are depicted in the first column, aggregated absolute errors vs uncertainties in the second column, sparsification curves in the third column, and the distribution of the proportionality constant α in the fourth column.

Figure 2 depicts direct comparisons between individual (first column) and aggregated (second column) absolute errors vs uncertainties as well as sparsification plots (third column) and the distribution of the ratios between the absolute error and uncertainty α (fourth column). Further uncertainty metrics are reported in the Supporting Information. The sparsification curves are obtained by ordering the test data points by either the absolute error or the uncertainty and then obtaining the mean absolute error over a fraction of the data points. Without removing any data points (i.e., the fraction of removed data points equals zero), this yields simply the mean absolute error averaged over all test data points, all atoms, and all directions. By iteratively removing test data (the highest values of error or uncertainty are removed first), the mean absolute error over the remaining data is therefore lowered if the ordering corresponds to the absolute error (oracle order). If the model uncertainties show the exact same order as the absolute errors, then the area between the oracle curve and the sparsification curve is zero. A large area therefore indicates that the uncertainties are not ordered according to the absolute error.

For all data sets, we observe large areas in the sparsification plots for the individual force uncertainties showing that they do not correlate with the absolute error and cannot be used to order the test set according to the estimated error. In fact, as we have explored in the previous subsection, single-individual force uncertainties and errors cannot correlate for mathematical reasons. To obtain a direct correlation, uncertainties and errors have to be averaged over sets of atoms (here, the full molecule or structure) to make use of eq 3. Although the data sets have a different number of data points and a different number of atoms per data point, they all show a large improvement in correlation between error and uncertainty upon aggregation over all atoms in a data point. In all cases, an ordering of the predicted values according to their uncertainty corresponds nearly perfectly to the oracle ordering according to the true prediction error, and the distribution of α is narrow. Note that the distribution of α is not centered around the expected value of  anymore, since all models feature differing amounts of model bias, so that the error from model variance is only a small part of the overall prediction error. Moreover, the model error may not be Gaussian anymore, causing further deviations from eq 3. However, this only affects the magnitude of α (since a change in distribution changes its first moment) but does not impede the quality of correlation between the aggregated errors and uncertainties, as long as the error is still symmetric around zero, and the model still performs adequately for the data of interest (see the Supporting Information for cases with low model performance).

**Figure 3:**
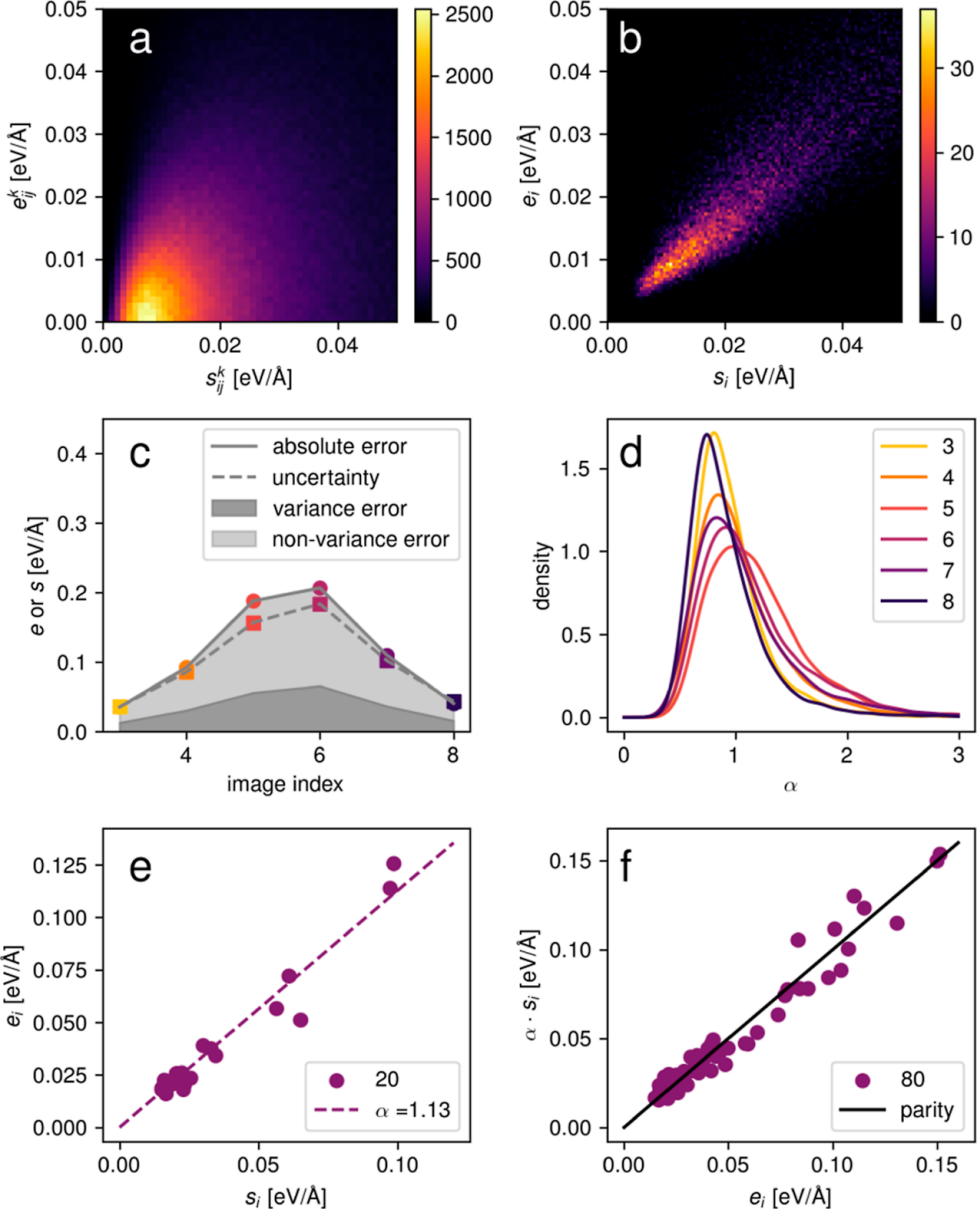
(a) Replot of the individual errors vs uncertainties as a heatmap for the Transition1x data set. (b) Replot of the molecular error vs uncertainty as a heatmap for the Transition1x data set. (c) Mean absolute error and mean uncertainty over all data points and all atoms as a function of the reaction path image index for Transition1x. (d) For each index, the distribution of α is plotted for the aggregated force uncertainties and errors. (e) Fit of α obtained from 20% of the SrTiO_3_ test set. (f) Prediction error obtained via the model uncertainty and the fitted value of α vs the true error for the remaining 80% of the test set.

Figure 3a,b furthermore depicts heatmaps of the individual and molecular errors vs uncertainties for the Transition1x data set, since the actual functional dependence is hard to read from Figure 2 due to the large number of test data points. Here, it becomes obvious that even for this difficult extrapolation task, the aggregation successfully leads to a highly correlated molecular error and uncertainty. The Transition1x data set is especially interesting to research the correlation between the error and uncertainty, since we can resolve it with respect to the NEB image index. Since indices 1, 2, 9, and 10 were used in the training and validation sets, we can plot the test set performance and uncertainty metrics over the indices 3, 4, 5, 6, 7, and 8. The indices 5 and 6 are the most dissimilar to the training set, which has never seen any transition-state structures but only (close-to) equilibrium structures. We therefore expect the model to have a much larger model bias for the indices 5 and 6 than the indices 3 and 8. Figure 3c depicts the mean absolute errors and mean uncertainty over all data points as functions of the index. We find that the model performs worst for indices 5 and 6, as expected. The model furthermore identifies these predictions as the most uncertain, featuring a high aggregated committee standard deviation. Furthermore, the model is able to identify a higher model bias (extrapolation error), Figure 3d, where the higher the values of distribution of α shift, the more the model encounters data points far away from the training set configurations. The values change from 0.93 for index 8 to 1.21 for index 5, as opposed to a pure-variance model with  (all values are given in the Supporting Information). Since eq 3 directly outputs the expected amount of variance error for a given data set and model architecture, we can furthermore compute the fraction of variance and nonvariance error of the overall error. The variance error is shaded in dark gray in Figure 3c, as well as the nonvariance error in light gray, which both visibly increase for structures far away from equilibrium. Together, the overall uncertainty strongly correlates with the actual error. The good correlation of aggregated uncertainties with aggregated errors for models with a significant amount of bias error may seem surprising, since technically the relation in eq 3 holds only for errors stemming from variance. Recently, the bias error was found to be correlated with the variance error when changing the data set size or model architecture.^29^ Here, we also find that the presence of model bias only changes the value of α and broadens its distribution slightly but preserves the correlation. In fact, we find that the bias and variance error are correlated for machine learning potentials (the variance error also increased for index 5 and 6), but the amount of bias error differs between the test sets with different degrees of extrapolation. To further illustrate the behavior of α, we also trained less-accurate versions of the presented MACE models and models based on the NeuralIL architecture.^2^ The results are compiled in the Supporting Information, together with correlation coefficients, mean α, and model bias percentages for all model types. Overall, the aggregated force uncertainties reliably identify sets of data points with a higher model bias (here, extrapolation error due to missing training data), which is an important prerequisite for successful active learning cycles.

Finally, we observe that although α never corresponds to the variance-only case, its true value can easily be obtained from a small test set and subsequently be used to transform between the model uncertainty (committee standard deviation) and the prediction error. Figure 3e depicts how α is fitted from 20% of the test data of the SrTiO_3_ system, which is then subsequently used to obtain the predicted error for the remaining 80% of the test data, Figure 3f. The predicted error is simply α*s*_*i*_ and can approximate the true error up to a mean absolute deviation of only 0.005 eV Å^–1^ for each single data point (for a model with a force mean absolute error of 0.042 eV Å^–1^), thus providing an accurate, quantitative proxy of the error without the need for a separate calibration step. This enables an easy and reliable identification of erroneous model predictions for a single data point, as opposed to a full data set. In the Supporting Information, we furthermore report predicted errors for the Transition1x data set resolved per NEB index.

### 3.3 Spatially Resolved Uncertainty

For large systems, we can take the aggregation concept one step further as we obtain sufficient statistics even when aggregating only over part of a large system instead of all atoms in the system. To obtain a spatially resolved error and uncertainty, we aggregate locally over *N*_*n*_ neighboring atoms *n* around atom *j* (including *j*) located within a cutoff *r*_cut_ via
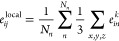
9

Similarly, the uncertainties can be averaged over all neighboring atoms as
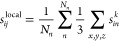
10Instead of a single absolute error and uncertainty per data point, we therefore obtain *N*_*j*_ local absolute errors and uncertainties for each data point, each centered around an atom *j*.

**Figure 4:**
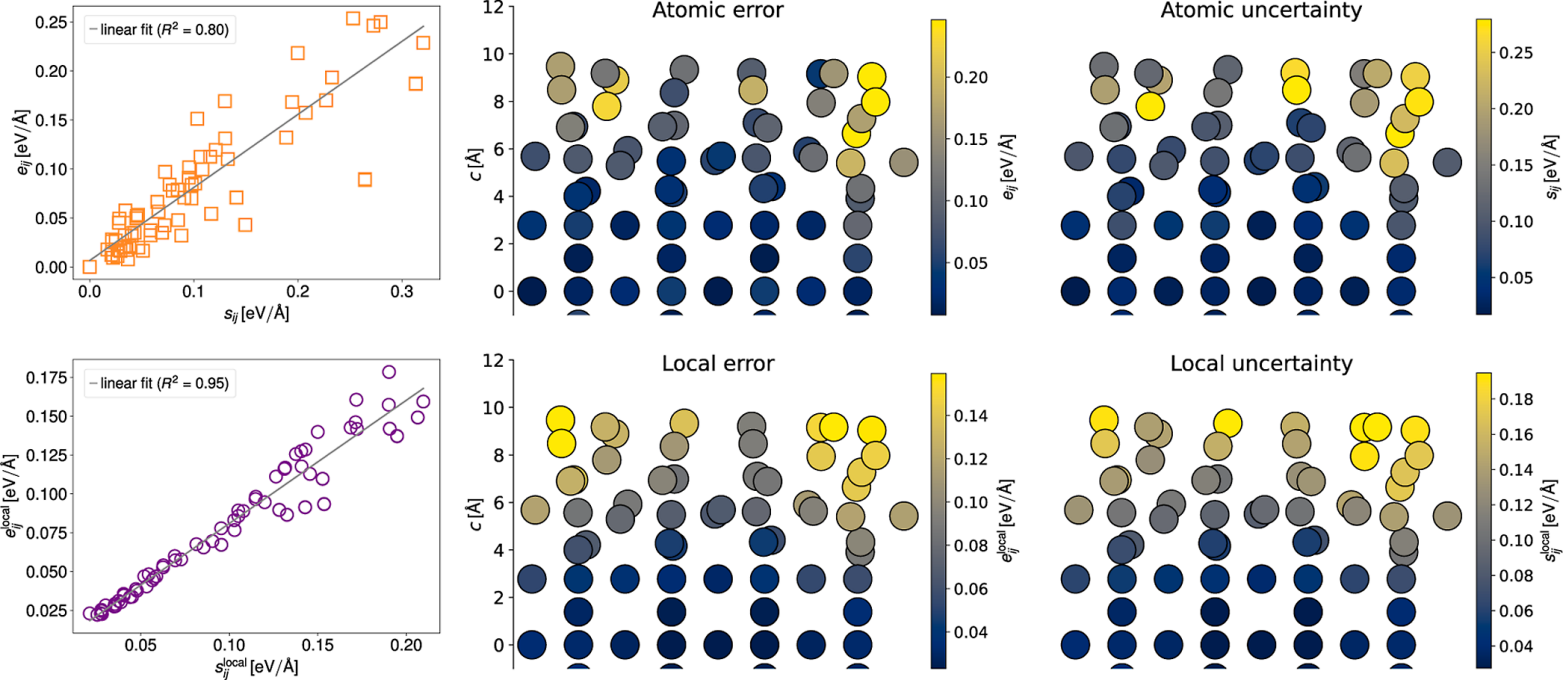
Two left-most panels show parity plots comparing uncertainty estimates to errors, atomic (top) and local (bottom), respectively. Spatially resolved errors and uncertainties for a data point from the SrTiO_3_ data set (side view) are illustrated in the other four panels. Atomic error and committee uncertainty obtained by summing over the three spatial directions for each atom (top). Local error and uncertainty, eqs 9 and 10, aggregated up to *r*_cut_ = 4 Å (bottom).

Spatially resolved errors and uncertainties for the SrTiO_3_ surface are shown in Figure 4, where a lighter color corresponds to a larger error or uncertainty. It is clear that mere atomic errors and uncertainties are not correlated, and the uncertainties provide a large number of false positives for the expected error. This is in agreement with earlier findings where the aggregated uncertainty of the entire system was used for an active learning procedure, because the atomic uncertainties failed to correlate with the error.^31^ In contrast, the local errors and uncertainties correlate very strongly so that the local uncertainty can be used to reliably identify high-error regions.

**Figure 5:**
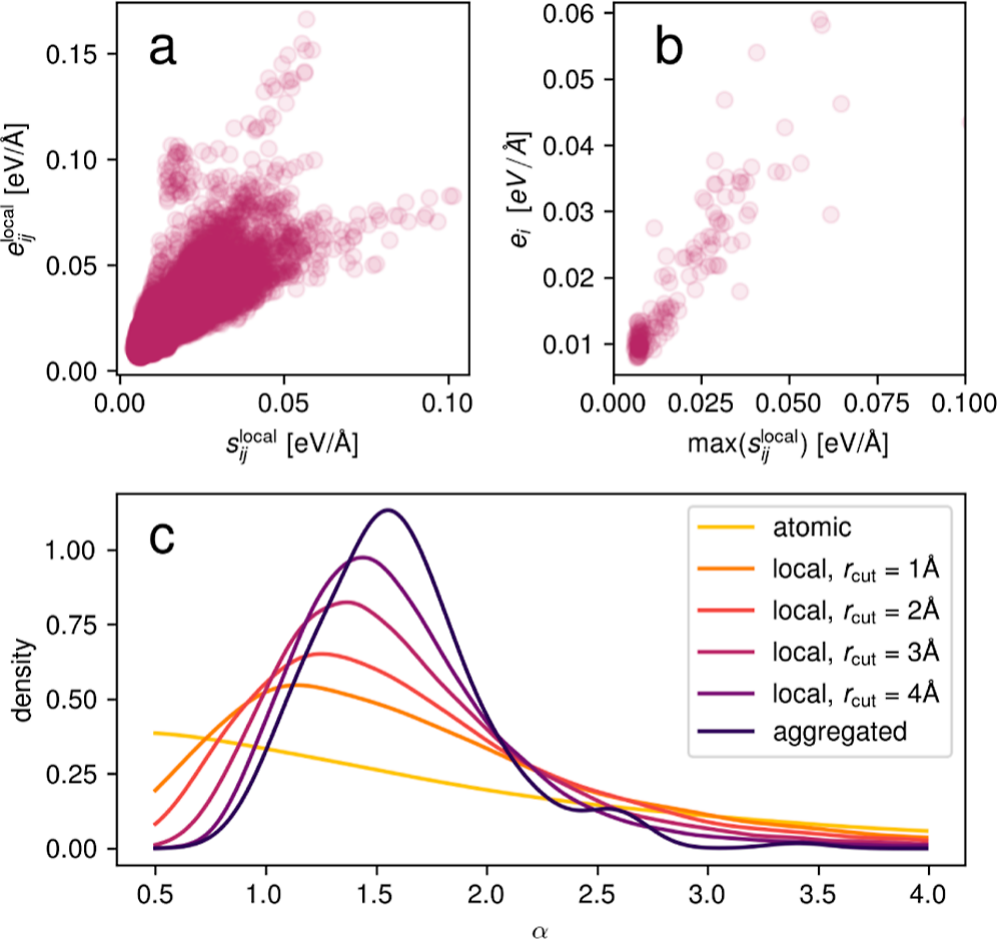
Local errors for the liquid water data set. (a) Local mean absolute error vs local uncertainty for *r*_cut_ = 5 Å. (b) Global mean absolute error for all atoms in a data point over the maximum of local uncertainties. (c) Ratio of the local error vs local uncertainty for different cutoff radii.

Figure 5a depicts the local mean absolute error vs the local uncertainty for *r*_cut_ = 5 Å for the liquid water data set. In contrast to the individual force component errors and uncertainties in Figure 2c which feature little correlation, the local errors and uncertainties are correlated. Furthermore, the maximum local uncertainty, Figure 5b, correlates with the overall mean absolute error averaged over all atoms in the data point, so that the locally aggregated force uncertainties can both be used to select high-error local substructures within a data point but also high-error data points. Figure 5c shows the distribution of the ratio of the local error vs local uncertainty for different cutoff radii. Again, it is seen that the atomic uncertainties are uncorrelated with the corresponding absolute error. In a molecular system such as water, one might intuitively aggregate over individual water molecules. Figure 5c shows that even such a highly localized aggregation, *r*_cut_ = 1 Å, leads to a certain degree of correlation. For larger cutoff radii, the correlation becomes stronger, and the locally aggregated uncertainty and error naturally converge to the globally aggregated quantities eventually. The choice of cutoff radius thus presents a trade-off between the quality of the correlation and the locality of the obtained values.

In the Supporting Information, we report detailed uncertainty metrics comparing atomic, local, and per-structure errors and uncertainties and find excellent correlation between the spatially resolved local errors and uncertainties developed in this study. The local uncertainties can be directly used to estimate local errors for the Transition1x, SrTiO_3_, and water data sets and feature high correlation coefficients, Spearman’s rank coefficients, as well as excellent error-based calibration. We furthermore note that the local uncertainties do not require a recalibration but only a simple fit of α to obtain direct error estimates via α*s*_*ij*_^local^.

In addition, the strong correlation between the local error and uncertainty makes the obtained uncertainties sharp, tight, and disperse. Sharpness quantifies the mean prediction uncertainty, where a method yielding high uncertainties across all predictions (low sharpness) is undesirable. Tightness refers to the small-scale reliability of uncertainty predictions with respect to reference values and is high for a perfect calibration.^34^ Dispersion describes the spread of observed uncertainties, where a model predicting the same uncertainty across all predictions (low dispersion) is undesirable.^52^ For all data sets and models reported, we observe sharp, tight, and disperse local uncertainty predictions due to their direct, strong correlation with errors.

### 3.4 Active Learning Using Spatially Resolved Uncertainty

**Figure 6:**
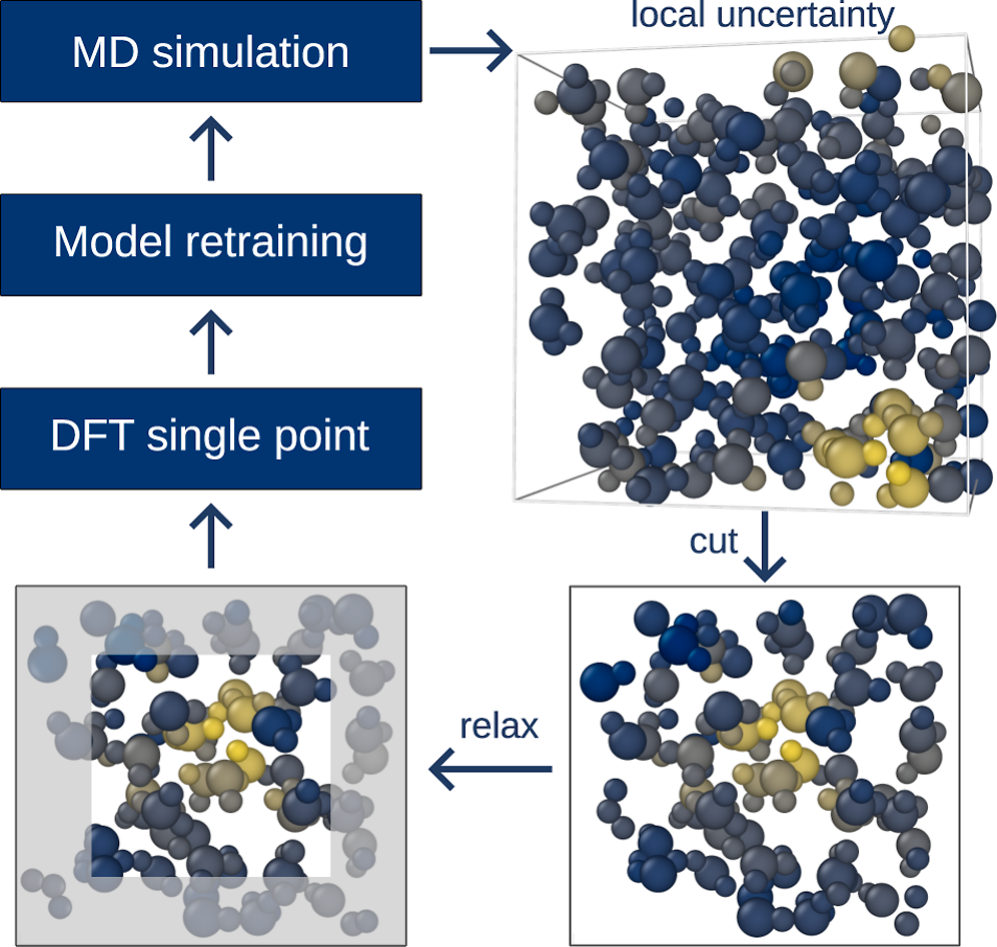
Schematic depiction of an active learning cycle, where an MD simulation with a machine learning potential generates a trajectory. Frames with high local uncertainties, indicated by lighter, yellow colors, are selected and cut into smaller boxes. The edge regions (gray background) are relaxed using the model and finally the energies and forces for the small boxes are then obtained ab initio and added to the training set, on which a new model is trained.

**Figure 7:**
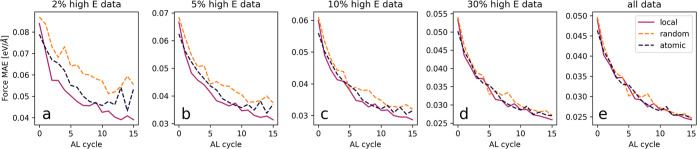
Model performance (mean absolute errors of the forces) as a function of the active learning cycle with each active-learning cycle adding ten data points of new ab initio calculations. The active learning is performed via a random (orange dashed lines), atomic uncertainty (blue dashed lines), or local uncertainty-based (purple full lines) sampling strategy. The different panels show different subsets of the test data, sorted by their potential energy.

With the spatially resolved uncertainties established as proxies for the local prediction error, we explore their use in an active learning scenario. The concept of local uncertainties is most helpful for large systems, where they would enable an active learning loop involving cutting out a fragment of the system for ab initio calculations instead of recomputing the full system. MD simulations pose an important application where a large simulation cell prohibits the addition of new training data of the full system. Here, we can make use of the direct correlation of local uncertainty with local error to design an active learning framework based on local subregions, where a model prediction is identified to fail. Similar approaches have been proposed before but suffer from selection based on atomic uncertainties,^35,39−41^ which we show to not correlate with the actual error. Furthermore, the scheme proposed in ref (39) only makes use of the forces on the central atom, thus not making use of a large amount of reference data. As detailed in the Methods section, we start with only 50 high energy water structures largely irrelevant to the density and temperature of interest. The active learning cycle is schematically depicted in Figure 6. The procedure consists of iteratively selecting subregions of the large simulation cell exhibiting large local uncertainty and constructing new small cells for which ab initio calculations are performed. Thereby, new training data are only added for the relevant subregions. Figure 7 depicts the performance of the uncertainty-based models on test sets also constructed from MD simulation (see the Methods section for details). For all test sets, the error falls off rapidly as data from the local substructures are added to the training set, with each active-learning cycle adding only ten data points. As comparison, we furthermore randomly selected frames and regions to cut out boxes, as well as based on the atomic, nonaggregated uncertainty. Averaged over all test data points, the accuracy of predicting forces does not differ significantly between the three approaches, because with the little amount of training data available, the addition of any data is helpful to the model. However, the uncertainty-sampling method largely outperforms random and atom-uncertainty sampling for high-energy structures even after only a few active learning cycles. This is especially important for MD simulations, where even infrequent wrong predictions of the forces can cause the trajectory to deviate from an ab initio trajectory significantly, or can cause the simulation to crash. We therefore largely favor a model not only able to predict typical configurations well but also extrapolate outside of that realm. The identification of ill-predicted environments via the local uncertainty therefore enables an efficient, fast, and effective way to collect new training data in an active learning scheme for MD simulation and yields stable models better able to extrapolate.

## 4 Conclusions

We have shown that the epistemic uncertainty of a single prediction obtained via a committee standard deviation cannot be directly correlated to the absolute error, reiterating the well-known asymmetric relation between error and uncertainty.^34^ For machine learning potentials, this holds true for both energy and atomic force predictions. Building on these results, we developed an approach to nevertheless use force uncertainties to identify high-error data points by aggregating force uncertainties and errors over groups of atoms. For variance-dominated models, a strong correlation between aggregated uncertainties and errors can be proven mathematically. For machine learning potentials, we further find that the approach holds for models containing a substantial amount of bias. For such models, we observe that the presence of bias amplifies the proportionality factor between aggregated uncertainties and errors compared with the variance-only case. Since the proportionality factor needs to be obtained from a fit to a validation set for any real-world scenario anyway, this does not impede the reliability of the proposed method. The aggregated force uncertainty is directly correlated both with the aggregated absolute error over that group of atoms but also the total absolute error of the model prediction for various data sets. Our approach confidently identifies high-error data points for systems with low and high numbers of atoms and is applicable to periodic and nonperiodic systems in the gas, liquid, and solid phase alike. We furthermore demonstrated that an aggregation must not necessarily include all atoms of a structure but can be restricted to neighboring atoms around an atom of interest. Locally aggregated uncertainties can then be applied to identify high-error local substructures and thus resolve absolute errors on an atomic scale. The benefits of locally aggregated uncertainties were showcased for an active learning study, indicating that data selection via spatially resolved uncertainties allows for data-efficient and fast training of accurate machine learning potentials. We therefore envision this workflow to be very powerful for active learning frameworks in low-data regimes for large, demanding systems, such as multiphase systems.

We furthermore note that the generality of our approach allows for application to a variety of systems and have already utilized spatially resolved uncertainties as developed in this study for surface reconstructions,^53^ interfaces, and defected structures (results are not yet published). These types of configurations consist of substructures featuring higher prediction errors than the rest of the structure, which is well resolved by the demonstrated approach. We therefore envision the current study to spark new applications within machine learning potentials, especially active learning cycles, across a large variety of systems.

## Data Availability

The Transition1x, SrTiO_3_, and water data sets including all data generated during the active learning loops for water are available on Zenodo at 10.5281/zenodo.11086346, together with scripts to calculate locally aggregated uncertainties and cut and relax water boxes for the active learning study, as well as a Jupyter notebook for the Monte Carlo experiment.

## Supporting Information Available

Additional benchmarks and exact uncertainty metrics for all systems (PDF)
