# Supplementary material for: Spatially Resolved Uncertainties for Machine Learning Potentials
Source: J Chem Inf Model. 2024 Aug 7;64(16):6377–87. doi: 10.1021/acs.jcim.4c00904 (PMC11351018; doi:10.1021/acs.jcim.4c00904)
Supplement: Supplementary file 1 — ci4c00904_si_001.pdf [file ci4c00904_si_001.pdf]

Supporting Information

Spatially Resolved Uncertainties for Machine Learning Potentials

Esther Heid,* Johannes Schörghuber, Ralf Wanzenböck, and Georg K. H. Madsen

Institute of Materials Chemistry, TU Wien, A-1060 Vienna, Austria

E-mail: esther.heid@tuwien.ac.at

Benchmark model

In the following, we revisit the Monte Carlo experiment from the main article, where the only model prediction error stems from variance. Fig. S1 depicts the observed conversion factors α between the prediction error and the uncertainty s or s_y with degrees of freedom 1 (default) or 1.5 (corrected estimator of the population standard deviation).[1] When taking the committee standard deviation s, the conversion factor depends on the number of committee members N_C. When taking the estimator of the standard deviation of the sample mean s_y (differing only by the factor $\sqrt{N_C}$), the conversion factor becomes independent of the number of committee members, and always equals $2/\sqrt{2\pi} \simeq 0.80$ consistent with Eq. 3. We note that both versions are correct, but always need to be denoted properly to avoid confusion. Since α does not have a physical meaning in itself, and can only be used together with the predictions of a committee to convert uncertainty to error, both versions create exactly the same predictions of model error. We furthermore note that the correction to the estimator

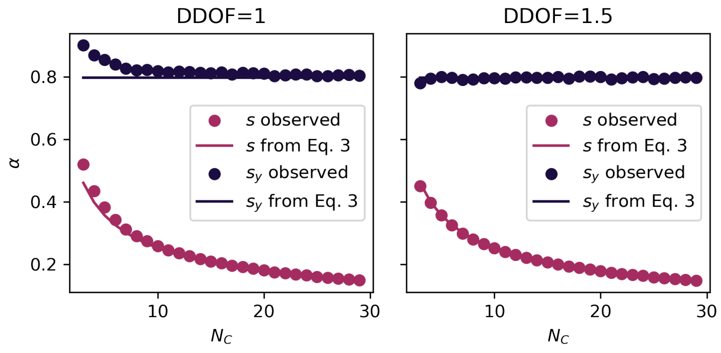

Figure S1: Dependence on the observed conversion factor between uncertainty and error as observed from s and s_y, compared to the theoretical values given by Eq. 3.

Table S1: Correlation between uncertainty and error as measured from R^2 of a linear model, as well as Spearman R.

	R^2				Spearman R			
System	Atomic XYZ	Atomic	Local	Structure	Atomic XYZ	Atomic	Local	Structure
Transition1x	0.39	0.53	0.69	0.69	0.69	0.86	0.93	0.93
- Index 3	0.41	0.55	0.77	0.77	0.56	0.77	0.93	0.93
- Index 4	0.37	0.51	0.66	0.65	0.64	0.82	0.91	0.91
- Index 5	0.34	0.48	0.44	0.41	0.65	0.82	0.70	0.68
- Index 6	0.35	0.47	0.42	0.40	0.68	0.85	0.68	0.65
- Index 7	0.32	0.45	0.56	0.55	0.62	0.80	0.84	0.83
- Index 8	0.35	0.51	0.69	0.70	0.50	0.72	0.88	0.88
$SrTiO_3$	0.40	0.64	0.89	0.96	0.53	0.70	0.89	0.93
Water	0.29	0.40	0.67	0.78	0.32	0.51	0.78	0.84

of the standard deviation of the population (with degrees of freedom 1 or 1.5) can become important for $N_C < 5$, to avoid substantial deviations from Eq. 3. Again, both versions can be used to compute α and thereby predict model errors from uncertainties, but need to be denoted correctly.

Uncertainty metrics for atomic, local, and per-structure errors

Table S1 lists correlation coefficients from a linear fit (R^2) and Spearman's rank correlation coefficients for all datasets between errors and uncertainties. The columns refer to errors and uncertainties that are either atomic and per spatial direction ("Atomic XYZ"), atomic but averaged over spatial directions ("Atomic"), averaged over local spheres with radius

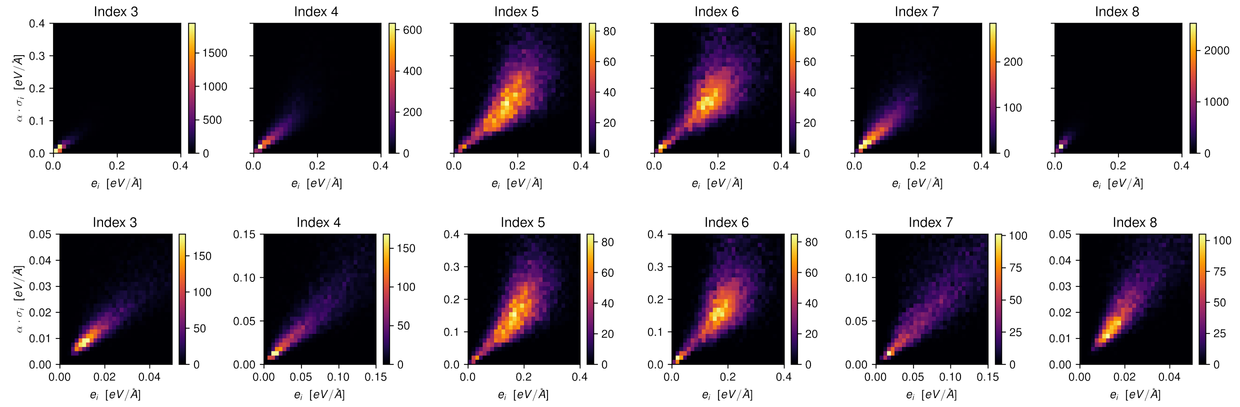

Figure S2: Parity plot (as heatmap, color corresponding to the bin count) of the predicted aggregated error (via α times the aggregated uncertainty) versus the actual aggregated error with shared axes (top), and zoomed-in axes (bottom) resolved per reaction index.

4 Å ("Local"), or averaged over the full structure of a data point ("Structure"). Clearly, the best correlation between error and uncertainty is achieved via averaging over all atoms within a data point, at the cost of losing atomic resolution for the error in the system. The local errors and uncertainties proposed in this study are nearly as well correlated, but give a spatially-resolved picture of the model performance within each data point. The local uncertainties and errors are much better correlated than atomic values, even when averaging the latter over spatial directions. Thus, the locally aggregated uncertainties proposed in this study are clearly better suited for tasks such as active learning than atomic or per-structure aggregated uncertainties. For the Transition1x data set, we furthermore repeated the analysis for the individual reaction indices, where index 3 and 8 are closest to the training set, and index 5 and 6 are furthest from the training set. For indices 3, 4, 7, and 8 we observe the same behavior as for the full data set, namely the local and per-structure uncertainties and errors being well correlated, while the atomic counterparts are not. For index 5 and 6, the correlation becomes poor regardless of the aggregation details, since the test data starts to deviate considerably from the training data. This demonstrates the limitations of ensemble uncertainty metrics in general: If the data of interest deviates too much from the training set, the ensemble uncertainty becomes increasingly unreliable to identify erroneous

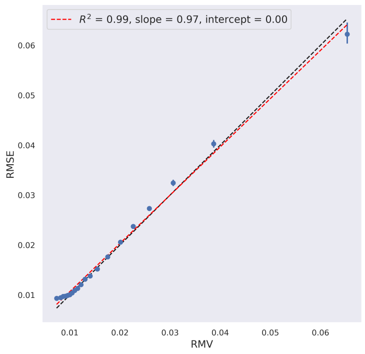

(a) Local, seed 59210631

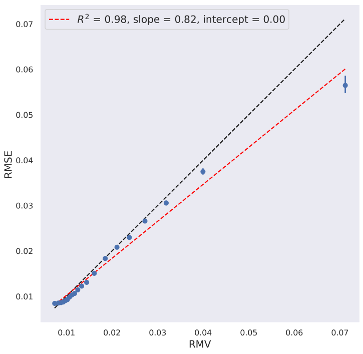

(b) Local, seed 1250918

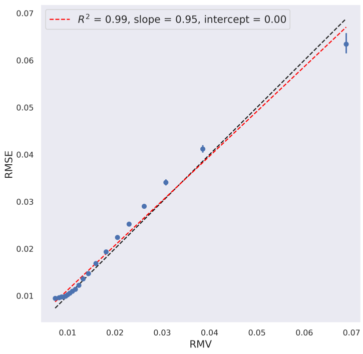

(c) Local, seed 219078156

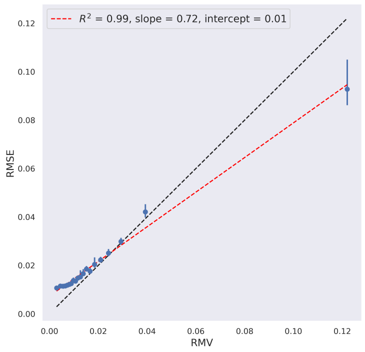

(d) Atomic, seed 59210631

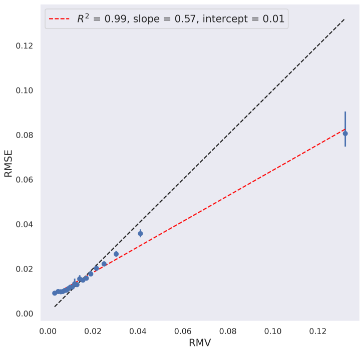

(e) Atomic, seed 1250918

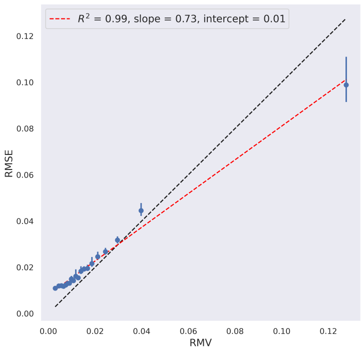

(f) Atomic, seed 219078156

Figure S3: Error-based calibration plots of local or atomic errors and uncertainties with different splitting seeds (in units of eV/Å), as obtained with the code by Ref. 2.

predictions. We therefore note that an adaptation of the training set may become important when encountering data far away from the training set.

We further explore the reliability of error prediction from aggregated committee uncertainty for Transition1x. Fig. S2 depicts a heatmap of the parity plot of the predicted versus actual aggregated error for the Transition1x dataset, resolved per reaction index. The value for α is fit from 100 (out of 10k data points) for each panel, the remaining 9.9k data points are plotted. The correlation coefficients range from 0.77 for index 3 to 0.40 for index 6, indicating that the correlation deteriorates the more the model needs to extrapolate, and thus misses data on the configurations of interest. We note that predicting energies and forces for index 5 and 6 is a very hard task with the model only being trained on (close to)

equilibrium structures (index 1, 2, 9, 10). Thus, the model never encountered large forces or nonequilibrium structures, to that even the low R^2 factor of 0.40 for index 6 is a rather good result.

For the water dataset, we furthermore computed the respective error-based calibration for the atomic and local errors and uncertainties (uncertainties scaled by α as obtained from a fit of 20% of the test data). Error-based calibration was initially proposed by Ref. 3, and identified to be a good uncertainty metric for chemical datasets by Ref. 2. Fig. S3 depicts the error-based calibration plots as obtained from the code provided with Ref. 2 for three different splitting seeds (between the 20% test data used for the fit of α and the remaining 80% test data used for plotting). For an ideally calibrated system, R^2 should be 1 with an intercept of 0 and a slope of 1. The local errors and uncertainties are nearly perfectly calibrated, while the atomic errors and uncertainties are not and feature a significantly too low slope, even though they were also rescaled with α (without rescaling, the slope is even worse).

High-bias models

In the main article, we trained large MACE models with a large size of hidden states and large cutoff radii. In the following, we present results similar to Fig. 2 for a two-layer MACE model with only 32 channels for even and odd parity features, and cutoff radius of 3.5 Å (in the following termed 'small MACE'), as well as for the invariant machine learning architecture NeuralIL.[4,5] MACE was trained as described in the main article. For $SrTiO_3$, NeuralIL was trained with Bessel descriptors computed with a cutoff radius of 4 Å and $n_{\max} = 5$, an energy and force loss weight of 0.1 and 0.9, respectively, with hidden layers 256:128:64:32:32:32:16 for 100 epochs. For the water data set, NeuralIL was trained with the same details for the Bessel descriptors and loss weights, but a smaller network of hidden layers 128:64:32:32 for 50 epochs. We furthermore tried to train NeuralIL models on Transition1x,

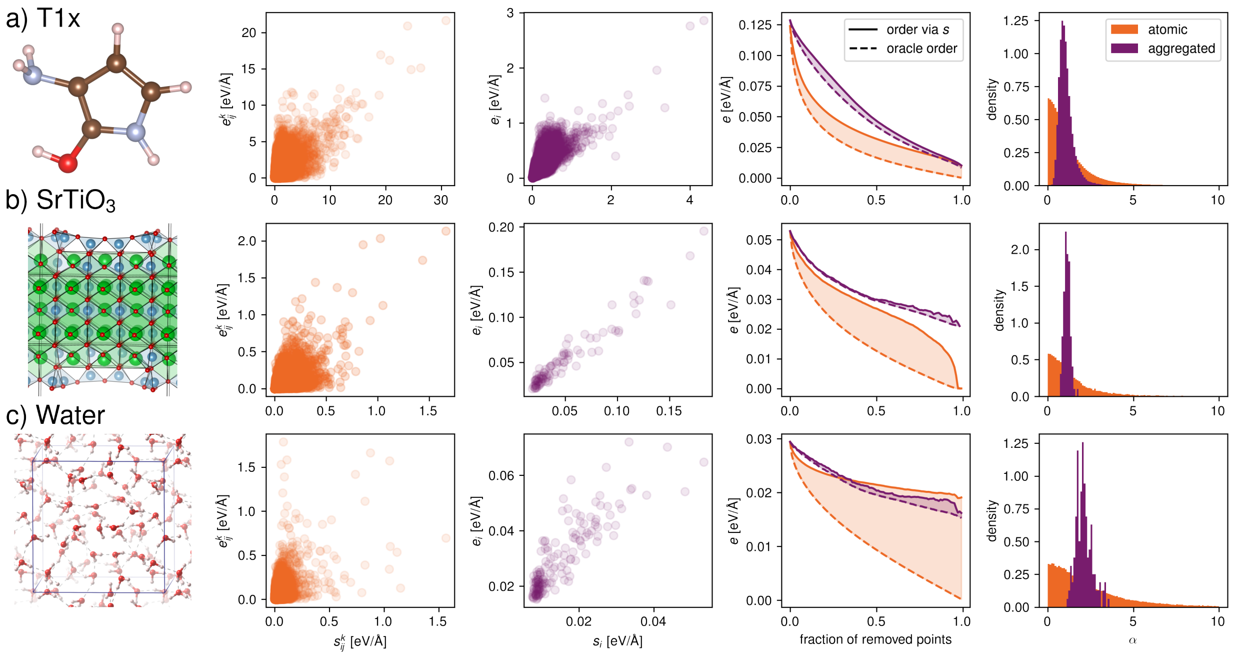

Figure S4: Relation between absolute error and uncertainty for small MACE models. Individual absolute errors versus uncertainties are depicted in the first column, aggregated absolute errors versus uncertainties in the second column, sparsification curves in the third column, and the distribution of the proportionality constant α in the fourth column.

however, this resulted in the force predictions to always be close to zero, since the limited architecture was not able to fit the diversity of structures observed in the dataset regardless of the hyperparameters. We therefore only report results for perovskite and water systems for NeuralIL.

Fig. S4 depicts the atomic uncertainty vs error, aggregated uncertainty vs error, sparsification curves, and histograms of the conversion factor alpha for the small MACE models. Fig. S5 depicts the same information for the NeuralIL committees (for the $SrTiO_3$ and water datasets). Compared to MACE in the main article, small MACE and especially NeuralIL features much higher error, stemming solely from model bias (with a less expressive architecture). This is directly visible in the magnitudes of errors, but also in the magnitude of conversion factors between error and uncertainty. Yet, the correlation of aggregated error and uncertainty still holds, as visible from the small areas in the sparsification curves, as well as the narrow ranges of α. Fig. S6 depicts the direct comparison between conversion

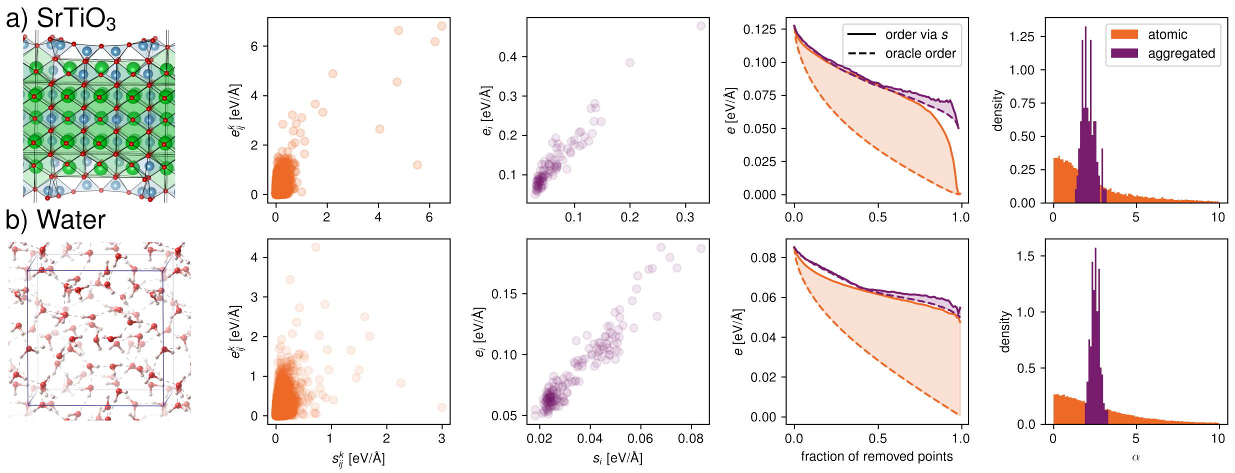

Figure S5: Relation between absolute error and uncertainty for NeuralIL models. Individual absolute errors versus uncertainties are depicted in the first column, aggregated absolute errors versus uncertainties in the second column, sparsification curves in the third column, and the distribution of the proportionality constant α in the fourth column.

Table S2: Mean absolute error, correlation coefficients, mean ratio between error and uncertainty, as well as percentage of model bias in the overall observed error for different models trained on different datasets. Arrows in parentheses indicate whether a high or low value is best.

System	Model	MAE force [eV/Å] (↓)	R^2 aggregated (↑)	$\overline{\alpha}$ (↓)	model bias
Transition1x	MACE	0.108	0.69	1.07	67%
	small MACE	0.124	0.70	1.08	67%
	NeuralIL	-	-	-	-
$SrTiO_3$	MACE	0.042	0.96	1.15	69%
	small MACE	0.053	0.94	1.12	68%
	NeuralIL	0.127	0.90	2.11	83%
Water	MACE	0.018	0.78	1.62	78%
	small MACE	0.029	0.75	2.10	83%
	NeuralIL	0.085	0.91	2.52	86%

factors of aggregated error and uncertainty for MACE, small MACE, and NeuralIL for the water dataset. Again, we observe that the aggregated properties are still nicely correlated despite the higher error from model bias, just with a larger value for α. The rise in α stems directly from the magnitude of model bias, and can thus be used to monitor the amount of bias present in the system.

Table S2 reports the mean absolute error of the predicted versus true atomic forces, the R^2 score of a linear model predicting aggregated errors from aggregated uncertainties, as well as the mean conversion factor (which also corresponds to the slope of the linear model). The expected α from variance only is 0.36, which we can convert to the amount of model

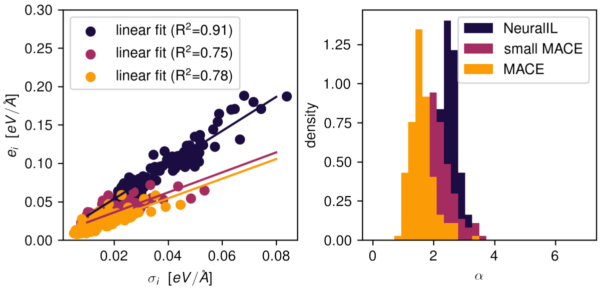

Figure S6: Relation between aggregated absolute error and uncertainty for the water dataset for MACE, small MACE, and NeuralIL models (left), as well as comparison between the distribution of the proportionality constant α (right). A worse and more restricted the architecture causes a larger proportionality constant α, since more of the actual error stem from model bias.

bias by comparing the actually observed value of α with 0.36. We directly observe how a smaller, less expressive models drives up the prediction error, as well as the conversion factor α. Yet, the correlation scores for the aggregated errors and uncertainties only suffer slightly when reducing model quality. The effect of bias thus averages out to some extent when aggregating over atomic errors and uncertainties, so that the principles described in the main article remain feasible even for higher bias models. We note though that there are limits to this observation, namely when a model architecture fails to fit a system, as observed for NeuralIL trained on the Transition1x dataset, where are forces are always predicted close to zero. Since all committee members follow this behavior, the uncertainty obtained from the committee disagreement is too small to capture the actual error.

References

(1) Brugger, R. M. A Note on Unbiased Estimation of the Standard Deviation. *Am. Stat.* **1969**, *23*, 32–32.

(2) Rasmussen, M. H.; Duan, C.; Kulik, H. J.; Jensen, J. H. Uncertain of uncertainties? A comparison of uncertainty quantification metrics for chemical data sets. *J. Cheminform.* **2023**, *15*, 1–17.

(3) Levi, D.; Gispan, L.; Giladi, N.; Fetaya, E. Evaluating and Calibrating Uncertainty Prediction in Regression Tasks. *Sensors* **2022**, *22*, 5540.

(4) Montes-Campos, H.; Carrete, J.; Bichelmaier, S.; Varela, L. M.; Madsen, G. K. H. A Differentiable Neural-Network Force Field for Ionic Liquids. *J. Chem. Inf. Model.* **2021**, *62*, 88–101.

(5) Carrete, J.; Montes-Campos, H.; Wanzenböck, R.; Heid, E.; Madsen, G. K. H. Deep Ensembles vs Committees for Uncertainty Estimation in Neural-Network Force Fields: Comparison and application to active learning. *J. Chem. Phys.* **2023**, *158*, 204801.
